# Neural activity in the prelimbic and infralimbic cortices of freely moving rats during social interaction: Effect of isolation rearing

**DOI:** 10.1371/journal.pone.0176740

**Published:** 2017-05-01

**Authors:** Chihiro Minami, Tomoko Shimizu, Akira Mitani

**Affiliations:** Laboratory of Physiology, Department of Human Health Sciences, Graduate School of Medicine, Kyoto University, Kyoto, Japan; Technion Israel Institute of Technology, ISRAEL

## Abstract

Sociability promotes a sound daily life for individuals. Reduced sociability is a central symptom of various neuropsychiatric disorders, and yet the neural mechanisms underlying reduced sociability remain unclear. The prelimbic cortex (PL) and infralimbic cortex (IL) have been suggested to play an important role in the neural mechanisms underlying sociability because isolation rearing in rats results in impairment of social behavior and structural changes in the PL and IL. One possible mechanism underlying reduced sociability involves dysfunction of the PL and IL. We made a wireless telemetry system to record multiunit activity in the PL and IL of pairs of freely moving rats during social interaction and examined the influence of isolation rearing on this activity. In group-reared rats, PL neurons increased firing when the rat showed approaching behavior and also contact behavior, especially when the rat attacked the partner. Conversely, IL neurons increased firing when the rat exhibited leaving behavior, especially when the partner left on its own accord. In social interaction, the PL may be involved in active actions toward others, whereas the IL may be involved in passive relief from cautionary subjects. Isolation rearing altered social behavior and neural activity. Isolation-reared rats showed an increased frequency and decreased duration of contact behavior. The increased firing of PL neurons during approaching and contact behavior, observed in group-reared rats, was preserved in isolation-reared rats, whereas the increased firing of IL neurons during leaving behavior, observed in group-reared rats, was suppressed in isolation-reared rats. This result indicates that isolation rearing differentially alters neural activity in the PL and IL during social behavior. The differential influence of isolation rearing on neural activity in the PL and IL may be one of the neural bases of isolation rearing-induced behavior.

## Introduction

Sociability promotes a sound daily life for individuals. Comfortable social interaction with other individuals enhances quality of life and maintains the stability of communities. Reduced sociability is a central symptom of various neuropsychiatric disorders, including autism spectrum disorder [1], depression [2], and schizophrenia [3], and yet the neural mechanisms underlying reduced sociability remain unclear. Studies employing imaging techniques report that structural and functional anomalies are induced in the medial prefrontal cortex (mPFC) of patients with autism spectrum disorder [4,5], depression [6,7], and schizophrenia [8,9]. These reports suggest that the mPFC functions as one of the neural bases of sociability.

The mPFC is located in the ventromedial region of the frontal lobe in humans and rodents [10]. The mPFC in rats consists of several distinct subdivisions that have been associated with various functions. Lesion of the mPFC promotes coping behavior in animals exposed to anxiety-provoking surroundings [11] and increases the duration of time spent in social interaction [12]. Injection of cobalt chloride, an inhibitor of synaptic activation, reduces freezing in the fear conditioning test [13] and induces an antidepressant-like effect in the forced swimming test [14]. In the ventral part of the mPFC, the prelimbic cortex (PL) and infralimbic cortex (IL) have direct fiber projections to the amygdala, which is one of the critical structures for the expression of emotion. The PL projects to the basolateral nucleus and capsular part of the central nucleus of the amygdala, whereas the IL projects to the medial, basolateral, central and cortical nuclei of the amygdala [15]. The PL and IL have been proposed to exert distinct, sometimes opposing, influences over behavior. PL stimulation increases conditioned freezing, whereas IL stimulation decreases conditioned freezing [16]. Infusion of the PL with muscimol, a GABA_A_ receptor agonist, depresses fear responses, whereas infusion of the IL disrupts recall of the extinction memory [17]. The PL regulates reward-related decision making, whereas the IL supports habit behavior (the “PL-go/IL-stop” model) [18]. These reports suggest that the PL and IL exert distinct functions and play important roles in the neural mechanisms underlying sociability.

Isolation rearing in the early stages of life has been shown to alter the brain and behavior of animals. Isolation rearing in rats results in structural changes in the mPFC, such as reductions in volume [19,20], dendritic length, and spine density [21,22]. Isolation-reared rats show locomotor hyperactivity to a novel situation [23,24], impaired sensorimotor gating [25], and anxiety-like behavior [26–28]. These changes in isolation-reared rats have been proposed to parallel those observed in humans with schizophrenia [19,24,29]. Thus, isolation-reared rats have the potential to provide findings related to the neural mechanisms underlying reduced sociability.

We hypothesized that isolation rearing leads to neural changes in the PL and IL that would affect social interaction. The purpose of the present study was to measure neural activity in the PL and IL of rats during social interaction and to determine whether there is neural activity related to social behavior in these areas, and to investigate the influence of isolation rearing on this activity.

It is necessary to record neural activity in behaving animals in order to investigate the neural basis of social interaction. Multiunit recording is a common technique to record neural activity in behaving animals; however, the conventional technique requires that the animal be tethered to recording instruments with a cable of electrical wires. The cable restricts the social behaviors of the animal and causes undue stress. Furthermore, the cable is easily tangled with other animals’ cables during social interaction. To overcome these issues, we made a wireless telemetry system to record the neural activity of a pair of freely moving rats during social interaction.

## Materials and methods

### Ethics statement

The experiment protocol was approved by the Animal Research Committee of Kyoto University Graduate School of Medicine (Permit Number: Med Kyo 16013). All surgery was performed under anesthesia with a gas mixture of isoflurane, and the animals were sacrificed under deep anesthesia with an overdose of sodium pentobarbital. All efforts were made to minimize suffering.

### Subjects and surgery

Sixty-four male Sprague-Dawley rats born in our laboratory were raised with their dam (Japan SLC, Inc., Shizuoka, Japan). They were divided equally into 2 groups at postnatal day (P) 21 and were housed either in groups (group-reared rats) or singly (isolation-reared rats). The group-reared rats were housed continuously with their dam and littermates for a period of 3 weeks (P21–P42) (during pre-adolescent to mid-adolescent development) [30] and then housed in groups of 3 per cage (38 × 22 × 20 cm high) for a period of 2 weeks (P43–P56). Rats reach early adulthood at P56 [30]. The isolation-reared rats were housed singly in a cage (28 × 18 × 13 cm high) for 5 weeks (P21–P56). Both groups had food and water available *ad libitum* and were housed at a constant room temperature (25°C) and a reverse 12-h light/dark cycle (lights on at 20:00 h). The experiments were carried out during the dark phase of the cycle (between 09:00 and 18:00 h).

**Fig 1:**
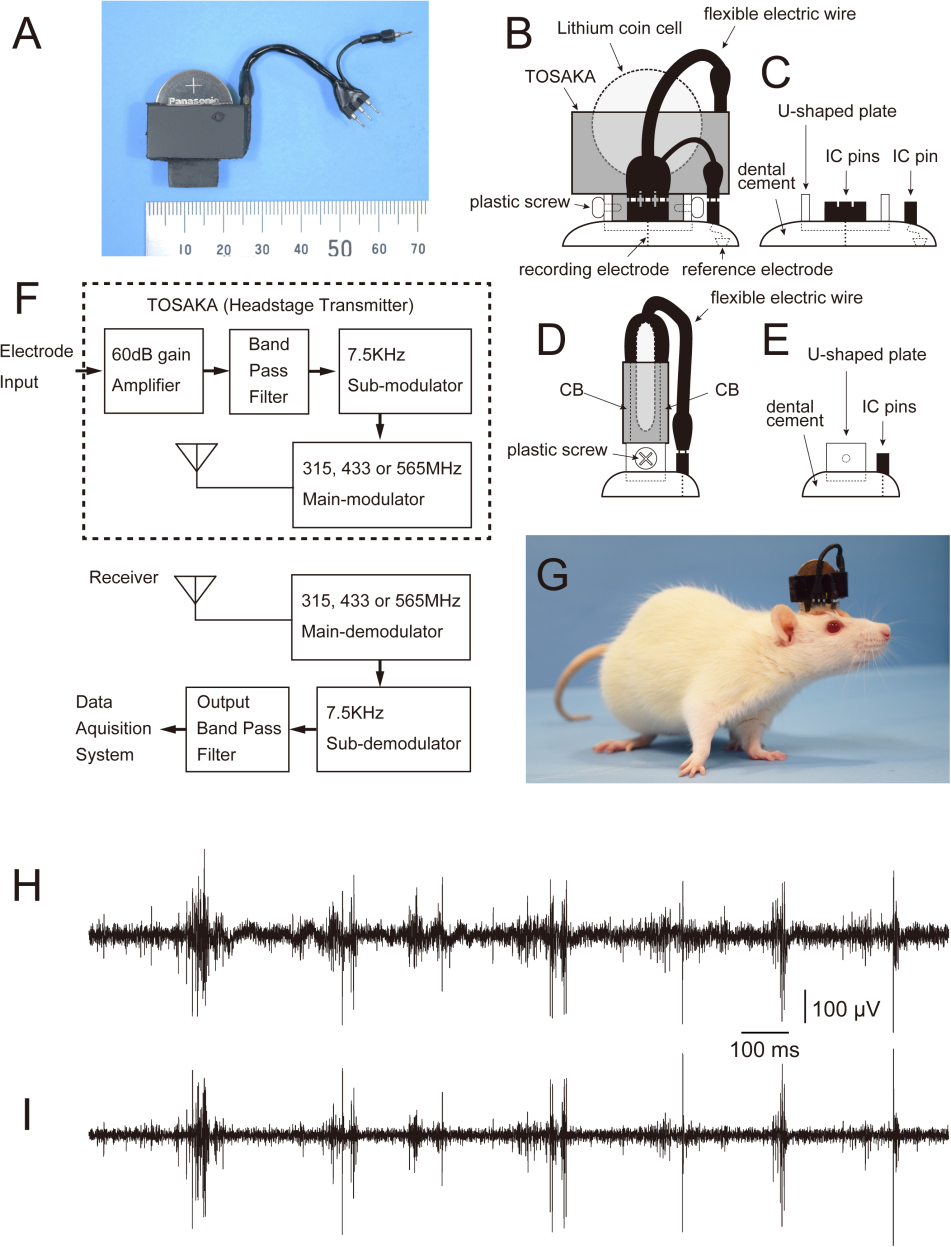
Overview of the wireless telemetry system. A: Wireless telemetry transmitter (Tosaka) that was designed to be attached to the head of a rat. It was 2.6 cm in length, 1.0 cm in width, and 1.9 cm in height, and it weighed 6.0 g. Transmitter range was approximately 10 m. A commercially available lithium coin cell battery (CR2032) was inserted into the Tosaka. Battery life was approximately 12 h. B: Drawing showing a Tosaka on the head of a rat (viewed from the right side). A Tosaka was attached into a U-shaped plate with plastic screws. A flexible electric wire was plugged into the Tosaka and IC pins that were connected to the recording electrodes. C: Drawing showing a block of dental acrylic cemented to the skull before attaching the Tosaka shown in (B). D: Drawing showing a Tosaka on the head of a rat (viewed from behind). CB: circuit board. E: Drawing showing a block of dental acrylic cemented to the skull before attaching the Tosaka shown in (D). F: System diagram of the transmitter and receiver. A neural signal was inputted into the input connector of a Tosaka, amplified with a gain of 1000, band-pass filtered (100–3000 Hz) with 3 pole band-pass filters, and modulated with a central frequency of 7.5 kHz by a voltage-controlled oscillator (sub-modulator). The signal was finally converted to a radio-frequency signal with a central frequency of 315, 433, or 565 MHz (main carrier frequency) by an RF modulator (main-modulator), and transmitted via an antenna. The radio frequency signal was received by 4 dipole antennas that were attached to the outside surface of a test box, demodulated by a main-demodulator, and then re-demodulated by a sub-demodulator. The signal was band-pass filtered (100–3000 Hz) and sent to a data acquisition system. G: Photograph of a rat equipped with a Tosaka. The Tosaka looks like a crest. A Tosaka with a flexible electric wire was usually wrapped with surgical tape for protection during the social interaction test. H, I: Neural activity was recorded from the PL in an anesthetized rat using a conventional tethered system (H) and the wireless telemetry system (I) simultaneously, in order to assess the wireless telemetry system. Comparison of the signals indicated that the signals obtained using the wireless telemetry system were comparable in quality to those acquired using the tethered recording system. Some differences were observed in spike waveforms and noise levels, which are thought to be mainly a result of differences in filtering and sampling.

On P57–P61, the rats (weight, 300–390 g) were unilaterally implanted with electrodes in the right PL or IL for multiunit recording during a social interaction test. The rats were anesthetized with a mixture of 2% isoflurane and nitrous oxide/oxygen (7:3) and placed in a stereotaxic instrument (Model 1430; David Kopf Instruments, Tujunga, CA). Body temperature was kept at 37°C with a heating lamp during the operative procedures. The scalp was incised and retracted, a hole was drilled at 11.0–13.0 mm anterior to the interaural line and 0.3–1.0 mm lateral to the midline, and the underlying dura was retracted carefully. A small hole was drilled in the nasal bone for a stainless steel screw that had been soldered to a flexible electric wire and used as a signal reference. The electric wire was connected to an IC pin (21501x4E; Linkman Co., Fukui, Japan). Three additional holes were drilled in the skull for stainless steel anchoring screws. Two urethane-insulated stainless-steel wires (50 μm in diameter, impedance of 140 kΩ; Unique-Medical Co., Tokyo, Japan) were used as recording electrodes. A straight array of the 2 recording electrodes (tip distance of approximately 100 μm) was lowered perpendicularly to either the PL (11.5–12.5 mm anterior to the interaural line, 0.6–0.8 mm lateral to the midline, and 2.0–2.5 mm ventral to the cortical surface) or IL (11.5–12.5 mm anterior to the interaural line, 0.5–0.7 mm lateral to the midline, and 3.7–4.5 mm ventral to the cortical surface) [31] using a micromanipulator (Model 1760-61SB; David Kopf Instruments) under an electrophysiological monitor of neural discharges, and cemented to the skull and anchoring screws with dental acrylic. The stainless-steel wire electrodes were connected to the IC pins. A U-shaped plastic plate, which was used to attach the wireless transmitter to the animal’s head, was cemented to the skull (Fig 1). All surgical incisions were sutured carefully. The rats were treated with antibiotics (benzylpenicillin; Meiji, Tokyo, Japan) and placed in a comfortable position on a warming blanket. After waking, the rats were returned to individual cages (38 × 22 × 20 cm high) in a recovery room and signs for potential pain and the surgical wounds were monitored for 7 days.

### Social interaction test and multiunit recording

The rats were allowed to recover for 7 days after surgery. The day (P64–P68) before the social interaction test, the rats were habituated to the test environment. The rats were anesthetized with a gas mixture of isoflurane. The wireless transmitter, which looked like a crest when it was attached to the head of a rat and we named it “Tosaka” (Tosaka means the crest of a cock in Japanese) (Fig 1), was attached to the U-shaped plate with plastic screws, and a flexible electric wire was plugged into both the Tosaka and IC pins connected to the electrodes. The Tosaka with the flexible electric wire was wrapped in surgical tape for protection. At approximately 1 h after recovering from the anesthesia, each rat was placed individually for 15 min in a test box located in a dark test room illuminated by red lighting (5 lux). The test box was made of opaque black Plexiglas (100 × 100 cm with walls 50 cm high). A video camera (Model CCD-2; Biotex Co., Kyoto, Japan) was fixed above the test box and relayed to a computer monitor in an adjacent room. At the end of each habituation session, the rats were moved from the test room. The Tosaka was detached from the head under anesthesia with a gas mixture of isoflurane, and the rats were returned to individual cages after waking. The floor and walls of the test box were cleaned with moistened tissue paper and dried.

The following day (P65–P69), each pair of rats underwent a social interaction test. Each group-reared rat was tested with a group-reared rat, and each isolation-reared rat was tested with an isolation-reared rat. The pairs of rats were novel to each other and did not differ by more than 20 g in weight. The rats were not used repeatedly. A Tosaka was attached to the head of one rat and other Tosaka with a different main carrier frequency was attached to the head of the partner in the same manner as on the previous day. The pairs of rats was placed at opposite corners of the test box, in which the back of one of the pair had been marked with black spray after the habituation session on the previous day, and the social interaction test was performed for 15 min. At the end of each social interaction test, the pair of rats was moved from the test room. The Tosakas were detached from each animal’s head under anesthesia with the gas mixture. The floor and wall of the test box were cleaned.

### Histology

After the social interaction test, all rats were anesthetized deeply with an overdose of sodium pentobarbital (100 mg/kg, i.v.) for marking the recording position and perfusion-fixation. The location of the tip of the recording wire electrode was marked by applying a positive current (1 μA, 60 s) through the electrode, and then the rats were perfused transcardially with saline followed by 10% formalin in 0.1 M phosphate buffer. Brains were removed and cryoprotected within 30% sucrose, and frontal frozen sections through the mPFC were cut at 50 μm on a sliding microtome. The marked position in the sections was visualized using a Prussian blue reaction. The sections were mounted onto gelatinized slides and counterstained with 0.3% neutral red.

### Data analysis

Behaviors in the social interaction test were categorized as “sole,” “approaching,” “contact,” and “leaving.”

1. Sole behavior: The rats behave separately from each other. Sole behavior includes resting (no movement), exploring (exploring around), rearing (standing on its hind legs and appearing to be looking at something), self-grooming (licking and biting its own fur and rubbing its forepaws over its head), and walking.
2. Approaching behavior: Approaching behavior includes “the rat approaching the partner” and “the partner approaching the rat.” The distance between both rats is reduced.
3. Contact behavior: Both rats are in contact with each other. Contact behavior includes social exploration (grooming, sniffing, and licking the partner’s body), aggressiveness (boxing, pinning, and biting the partner), and defensiveness (crawling under the partner and receiving the partner’s attack).
4. Leaving behavior: Leaving behavior includes “the rat leaving the partner” and “the partner leaving the rat.” The distance between both rats is increased.

Behavior was scored by two observers. Behaviors scored in each rat were the latency of the rat to first approaching behavior and the total duration and frequency of sole, approaching, contact, and leaving behavior. The average durations of sole, approaching, contact, and leaving behavior were calculated for each rat.

**Fig 2:**
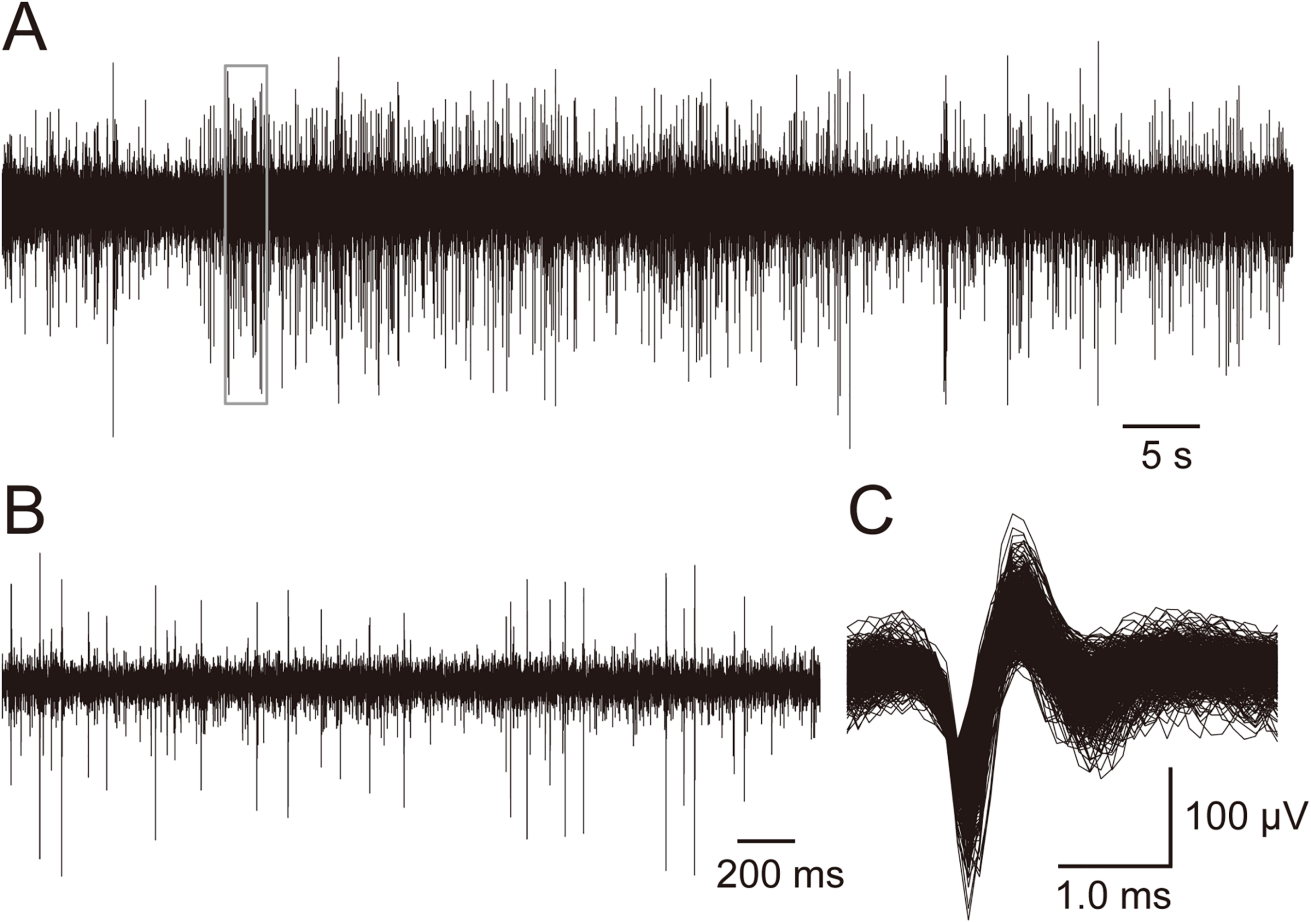
Wireless recording of multiunit activity in a freely moving rat. A: Raw neural activity recorded from the IL during the social interaction test. B: Close up view of the neural activity enclosed by a rectangle in (A). Multiunit activities with various amplitudes are observed. C: Multiunit activities extracted from the raw neural activity in (A). Multiunit activities with various amplitudes show similar waveforms, which suggests that all of the neurons recorded are the same type.

Neural activity was continuously acquired, sampled (10 kHz), and stored with a PowerLab/LabChart-7 system (ADInstruments, Nagoya, Japan). Data were analyzed off-line using custom-designed software with a template-based sorting program (Multiunit sorting, counting, and analyzing tools, ver. 8, Muscat-8) running on personal computers. Spikes with a signal-to-noise ratio > 2.0 were discriminated and counted (Fig 2). As the primary focus of this experiment was to investigate the responding populations of neurons in the PL and IL, rather than to analyze differences in the responses of single neurons, all recorded unit activity was analyzed as multiunit activity.

Results are expressed as the mean ± standard error of the mean (SEM). Student’s *t* test, paired *t* test, multiple comparison procedures, and one-way repeated measures analysis of variance (ANOVA) followed by a *post hoc* comparison were used as indicated in the text. The criterion for significance was *P* < 0.05 (SPSS Statistics 22.0; IBM, Tokyo, Japan).

## Results

### Group-reared rats

#### Behaviors

**Fig 3:**
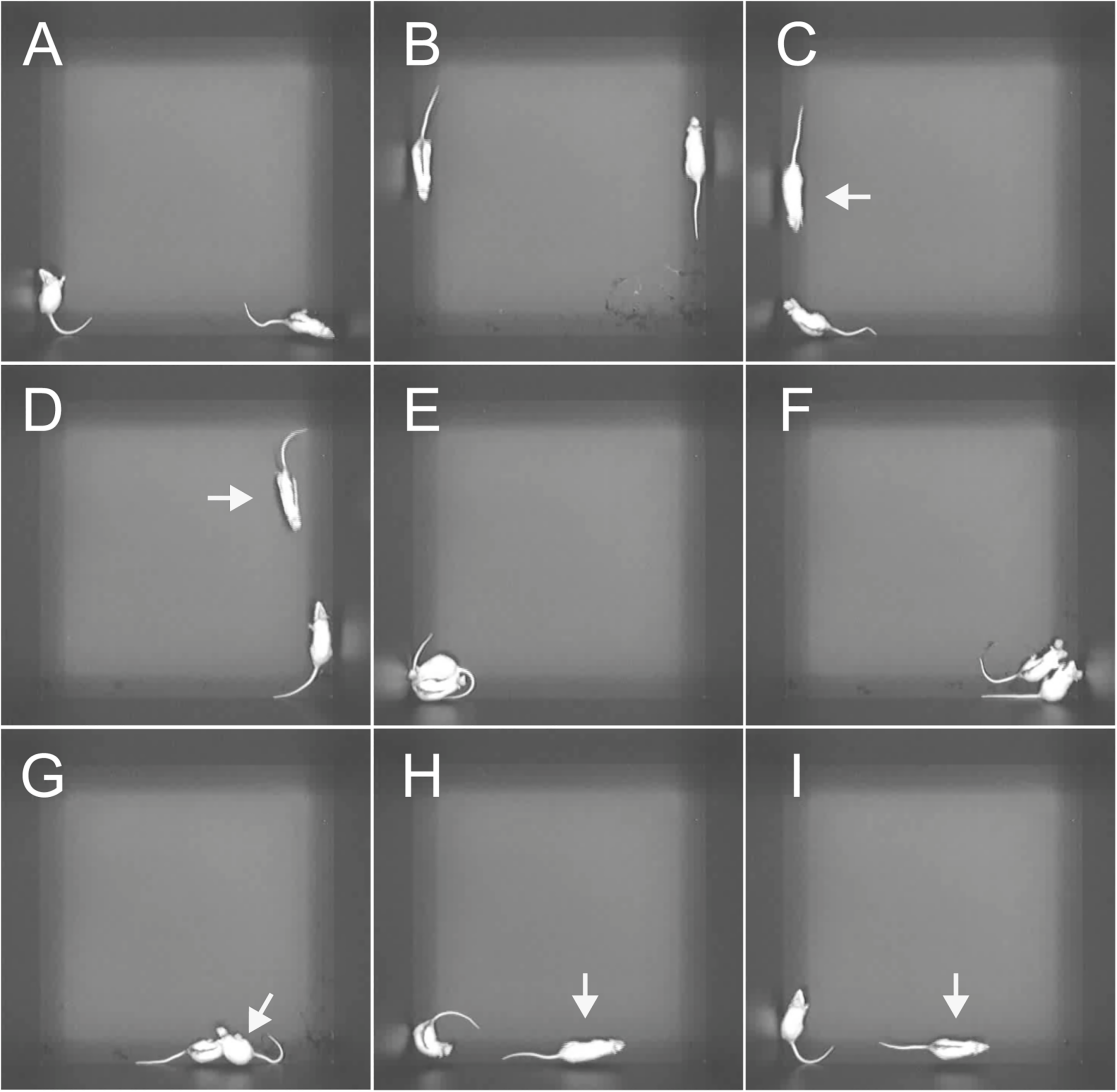
Examples showing the behavior of the rats during the social interaction test. A Tosaka was attached to each animal’s head. The back of one of the pair was marked with black spray. The pair of rats was placed in a test box located in a darkened room. A, B: Sole behavior. Rats are behaving separately. The rat (no mark on its back) is exploring around by itself at the lower left corner of the test box and the partner (a black mark on its back) is resting at the lower right corner (A). Both rats are walking along the wall (B). C, D: Approaching behavior. The rat (arrow) is approaching the partner (C), and the partner (arrow) is approaching the rat (D). E–G: Contact behavior. Rats are sniffing (E) and boxing (F) each other, and the rat (arrow) is pinning and biting the partner (G). H, I: Leaving behavior. The rat (arrow) is leaving the partner (H), and the partner (arrow) is leaving the rat (I).

After placement at opposite corners of the test box, the rats explored their environment (Fig 3A) and walked along the wall (Fig 3B). Then, they showed approaching behavior (Fig 3C and 3D). They reduced the distance between them and came into contact with each other. The mean latency until first approaching behavior was 19.2 ± 2.6 s. The mean total duration of approaching behavior was 27.1 ± 2.2 s, and the mean frequency of approaching behavior was 14.7 ± 1.2 (Table 1). The mean duration per one approaching behavior was 1.9 ± 0.1 s. During contact behavior, the rats engaged in social exploring behaviors (grooming, sniffing [Fig 3E], and licking the partner’s body), aggressive behaviors (boxing [Fig 3F], pinning, and biting the partner [Fig 3G]), or defensive behaviors (crawling under the partner and receiving an attack [Fig 3G]). One rat attacked the other and the other defended itself, and reversals in attacker and defender were not observed during an episode of aggressive and defensive behavior. The mean total duration of contact behavior was 466.6 ± 26.6 s, which was approximately half of the period of the social interaction test. The mean frequency of contact behavior was 14.4 ± 1.1. The mean duration per one contact behavior was 40.1 ± 4.6 s. Leaving behavior was observed at the end of contact behavior. The distance between both rats was increased, in which one of the rats left the partner and walked to the corner of the test box (Fig 3H and 3I) or both rats left each other and walked to a separate corner. The mean total duration of leaving behavior was 31.6 ± 2.5 s and the mean frequency of leaving behavior was 14.2 ± 1.2. The mean duration per one leaving behavior was 2.3 ± 0.1 s.

**Table 1:** Duration and frequency of behaviors of group-reared rats (n = 32) during the 15-min social interaction test.

	Total duration (s)	Frequency of episodes	Duration per episode (s)
Sole behavior	374.8 ± 25.6	15.1 ± 1.2	29.2 ± 3.0
Approaching behavior	27.1 ± 2.2	14.7 ± 1.2	1.9 ± 0.1
Contact behavior	466.6 ± 26.6	14.4 ± 1.1	40.1 ± 4.6
Leaving behavior	31.6 ± 2.5	14.2 ± 1.2	2.3 ± 0.1

Results are means ± SEM.

#### Multiunit activity

**Fig 4:**
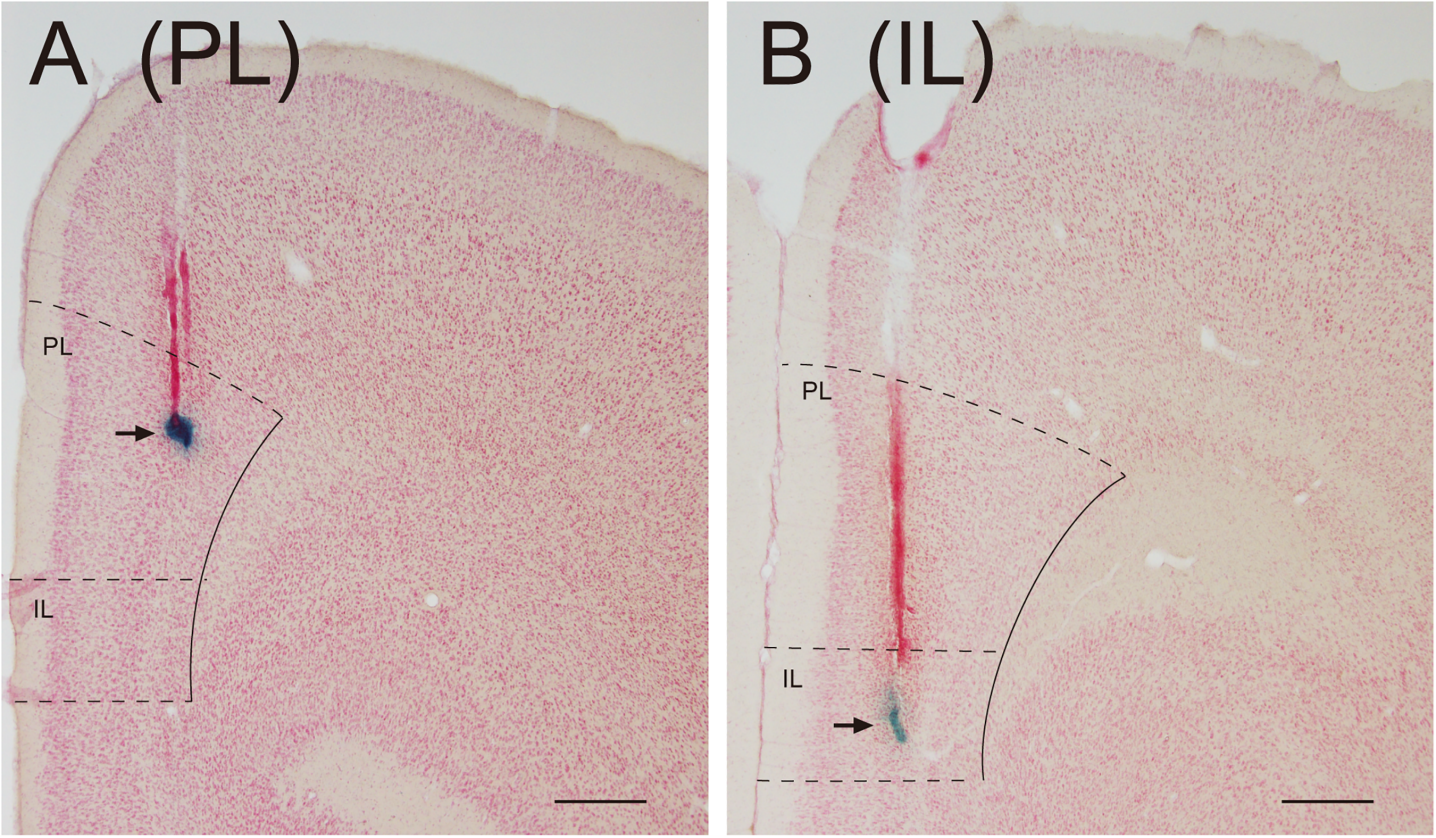
Photomicrographs showing the position of the recording sites. A, B: Arrows indicate the mark of the tip of a recording electrode in the PL (A) and IL (B). The tip of the recording electrode is identified with a blue spot of deposited iron. Outlines of the PL and IL are indicated with solid and dashed lines. Scale bars = 0.5 mm.

Multiunit activity of the PL was recorded in 15 of 16 rats during the social interaction test; all recording sites were located in the PL (Fig 4A), but clear multiunit activity (signal-to-noise ratio > 2.0) was not recorded from 1 rat. Multiunit activity of the IL was recorded in 12 of 16 rats during the social interaction test; recording sites were located in the IL in 14 rats (Fig 4B), and clear multiunit activity was not recorded from 2 of the 14 rats.

**Fig 5:**
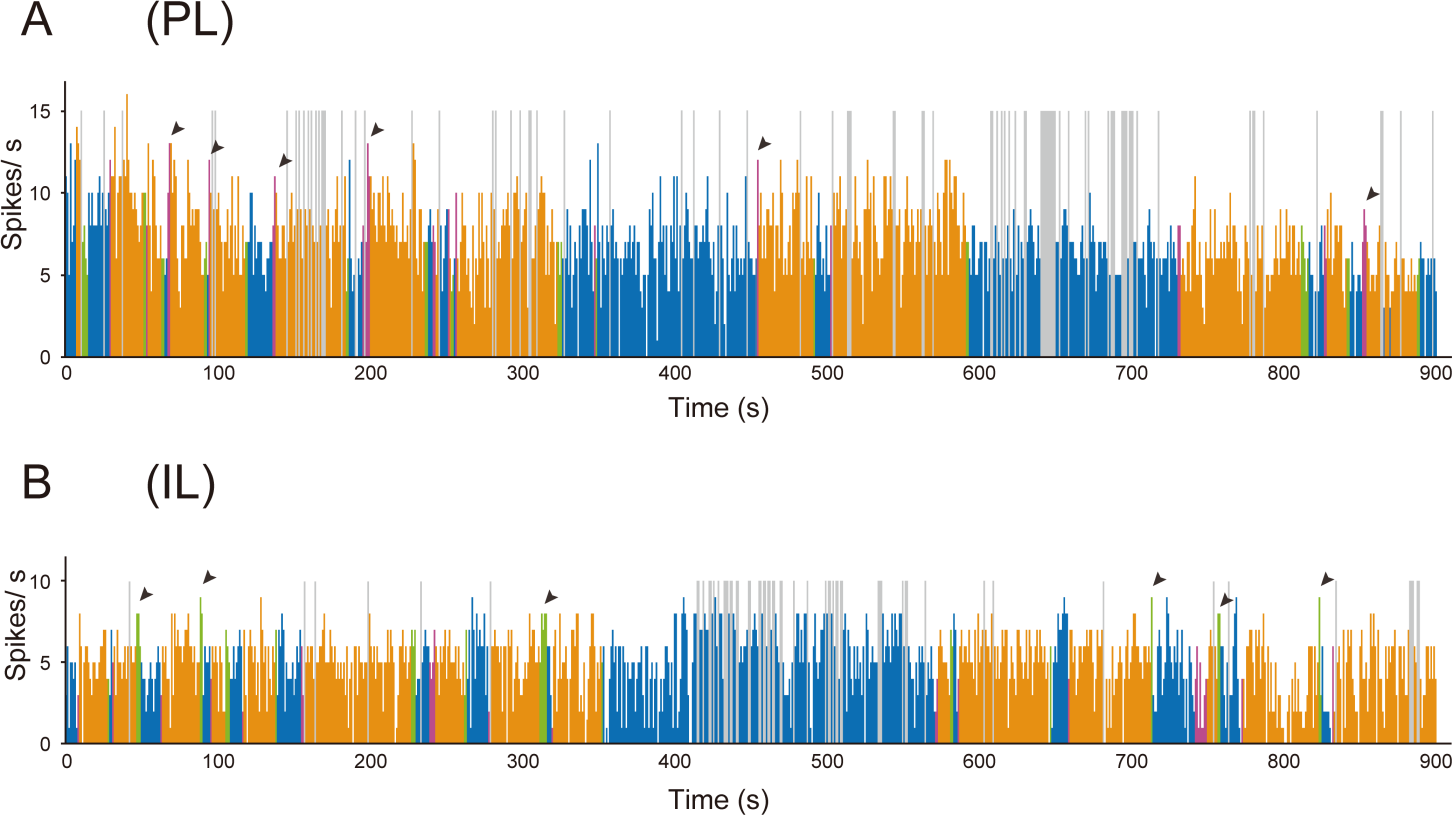
Histograms illustrating multiunit activity of PL and IL neurons during the social interaction test. A: Multiunit activity recorded in the PL. The blue, red, orange, and green columns represent firing rate during sole, approaching, contact, and leaving behavior, respectively. The firing rate increases during approaching (red columns) and contact (orange columns) behavior. The arrowheads indicate some of the instances when increased firing rates were observed during approaching behavior. Mean firing rates during sole, approaching, contact, and leaving behavior in this rat were 6.5 Hz, 9.0 Hz, 7.5 Hz, and 6.4 Hz, respectively. B: Multiunit activity recorded in the IL. The blue, red, orange, and green columns represent firing rate during sole, approaching, contact, and leaving behavior, respectively. The firing rate increases during leaving behavior (green columns). The arrowheads indicate some of the instances when increased firing rates were observed during leaving behavior. Mean firing rates during sole, approaching, contact, and leaving behavior in this rat were 5.0 Hz, 3.4 Hz, 4.9 Hz, and 6.4 Hz, respectively. The histogram bin size is 1 s. The gray columns indicate that multiunit activity was not discriminated from the artificial noise generated by sharp head shaking and self-grooming. When more than 2 behaviors were observed in a period of 1 column, the color of the behavior observed for the longest time is applied to the column.

Fairly stable firing was observed during sole behavior in PL and IL neurons (blue columns in Fig 5). Firing was observed to increase during approaching behavior in PL neurons (red columns in Fig 5A), but not in IL neurons (red columns in Fig 5B). During contact behavior, an increase in firing was observed in PL neurons (orange columns in Fig 5A), but not in IL neurons (orange columns in Fig 5B). During leaving behavior, an increase in firing was not observed in PL neurons (green columns in Fig 5A), but it was observed in IL neurons (green columns in Fig 5B).

**Fig 6:**
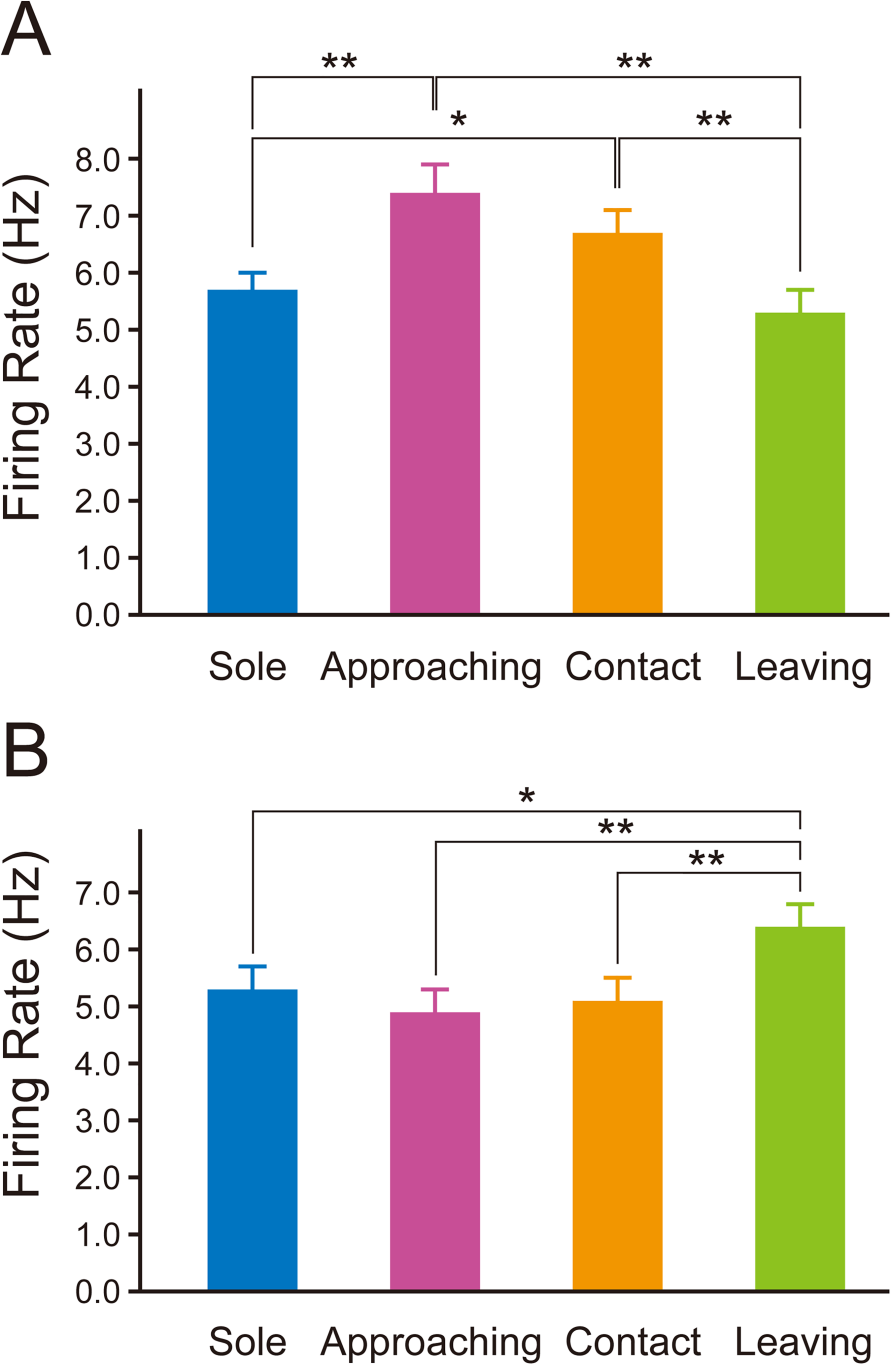
Firing rate of PL and IL neurons in the social interaction test. A, B: Mean firing rates of PL neurons (n = 15) (A) and IL neurons (n = 12) (B) during sole (blue columns), approaching (red columns), contact (orange columns), and leaving (green columns) behavior. The firing rates of PL neurons during approaching and contact behavior were significantly higher than during sole and leaving behavior (A). The firing rate of IL neurons during leaving behavior was significantly higher than during sole, approaching, and contact behavior (B). Data represent means ± SEM. *P < 0.05, **P < 0.01; one-way repeated measures ANOVA with Bonferroni *post hoc* test.

In the 15 rats in which multiunit activity was recorded in the PL, the mean firing rate during sole behavior was 5.7 ± 0.3 Hz (range: 3.2–7.6 Hz). In the 12 rats in which multiunit activity was recorded in the IL, the mean firing rate during sole behavior was 5.3 ± 0.4 Hz (2.2–7.6 Hz). The mean firing rates of PL and IL neurons during approaching behavior were 7.4 ± 0.5 Hz (4.4–10.2 Hz) and 4.9 ± 0.4 Hz (2.2–6.7 Hz), respectively. The mean firing rates of PL and IL neurons during contact behavior were 6.7 ± 0.4 Hz, (4.2–9.5 Hz) and 5.1 ± 0.4 Hz (2.8–8.6 Hz), respectively. The mean firing rates of PL and IL neurons during leaving behavior were 5.3 ± 0.4 Hz (2.7–8.2 Hz) and 6.4 ± 0.4 Hz (4.1–10.1 Hz), respectively. One-way repeated measures ANOVA revealed a significant difference among the firing rates of PL neurons during the 4 types of behavior (F_(3,42)_ = 14.60; *P* < 0.001). *Post hoc* comparisons (Bonferroni test) showed that the firing rate during approaching behavior was significantly higher than during sole behavior (*P* = 0.001) and leaving behavior (*P* < 0.001) and also that the firing rate during contact behavior was significantly higher than during sole behavior (*P* = 0.011) and leaving behavior (*P* = 0.001) (Fig 6A). There was no significant difference between the firing rates during approaching behavior and contact behavior (*P* = 0.064) and also between the firing rates during sole behavior and leaving behavior (*P* = 0.275). One-way repeated measures ANOVA revealed a significant difference among the firing rates of IL neurons during the 4 types of behavior (F_(3,33)_ = 8.361; *P* < 0.001). *Post hoc* comparisons showed that the firing rate during leaving behavior was significantly higher than during sole behavior (*P* = 0.020), approaching behavior (*P* = 0.002), and contact behavior (*P* = 0.001) (Fig 6B). There was no significant difference between the firing rates during sole, approaching, and contact behavior (“sole” vs. “approaching,” *P* = 0.145; “sole” vs. “contact,” *P* = 0.639; and “approaching” vs. “contact,” *P* = 0.588).

**Fig 7:**
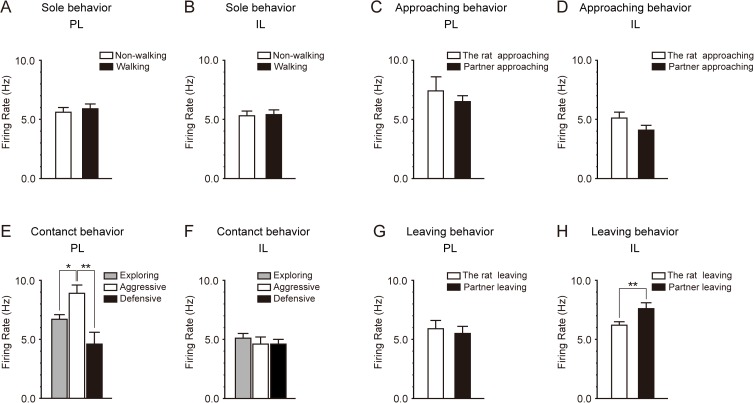
Firing rate of PL and IL neurons during sole, approaching, contact, and leaving behavior. A, B: The firing rate of PL (A) and IL (B) neurons during sole behavior. Sole behavior was classed into non-walking (white columns) and walking (black columns) behavior. There were no significant differences between the firing rates during non-walking and walking behavior. C, D: The firing rate of PL (C) and IL (D) neurons during approaching behavior. Approaching behavior was classed into “the rat approaching the partner” (white columns) and “the partner approaching the rat” (black columns). There were no significant differences between the firing rates when the rat approached the partner and when the partner approached the rat. E, F: The firing rate of PL (E) and IL (F) neurons during contact behavior. Contact behavior was classified into 3 groups: socially exploring (gray columns), aggressive (white columns), and defensive (black columns). The firing rate during aggressive behavior was significantly higher than during socially exploring and defensive behavior in PL neurons (E), whereas no significant change in firing rate was observed in IL neurons (F). G, H: The firing rate of PL (G) and IL (H) neurons during leaving behavior. Leaving behavior was classed into “the rat leaving the partner” (white columns) and “the partner leaving the rat” (black columns). There was no significant difference between the firing rates of PL neurons when the rat left the partner and when the partner left the rat (G). The firing rate of IL neurons when the partner left the rat was significantly higher than when the rat left the partner (H). Data represent means ± SEM. *P < 0.05, **P < 0.01.

Sole behavior was classed into non-walking and walking behavior. Non-walking behavior included resting, exploring around, rearing, and self-grooming. During walking behavior, the rats usually walked along the wall. In the PL, the firing rates during non-walking behavior and walking behavior were 5.6 ± 0.4 Hz and 5.9 ± 0.4 Hz, respectively, which were not significantly different (*P* = 0.469, paired *t* test, n = 15) (Fig 7A). In the IL, the firing rates during non-walking behavior and walking behavior were 5.3 ± 0.4 Hz and 5.4 ± 0.4 Hz, respectively, which were not significantly different (*P* = 0.920, n = 12) (Fig 7B). These results indicate that the neural activity of PL and IL neurons was not directly related to locomotion.

In the 15 rats in which multiunit activity was recorded in the PL, 7 showed both types of approaching behavior, in which the rat approaches the partner and the partner approaches the rat. Four rats showed only the approaching behavior in which the rat approaches the partner, and the other 4 rats showed only the approaching behavior in which the partner approaches the rat. In order to verify whether the factor of which rat approaches was significant in the increase in firing rate during approaching behavior, statistical analysis was performed in the 7 rats that showed both types of approaching behavior. There was no significant difference between the firing rates of PL neurons when the rat approached the partner (7.4 ± 1.2 Hz) and when the partner approached the rat (6.5 ± 0.5 Hz) (*P* = 0.323, paired *t* test, n = 7) (Fig 7C). This result indicates that the factor of which rat approaches was not significant in the increase in the firing rate of PL neurons during approaching behavior. In the 12 rats in which multiunit activity was recorded in the IL, 10 showed both types of approaching behavior. One rat showed only the approaching behavior in which the rat approached the partner, and another rat showed only the approaching behavior in which the partner approached the rat. Statistical analysis was performed in the 10 rats that showed both types of approaching behavior. There was no significant difference between the firing rates of IL neurons when the rat approached the partner (5.1 ± 0.5 Hz) and when the partner approached the rat (4.1 ± 0.4 Hz) (*P* = 0.145, paired *t* test, n = 10) (Fig 7D).

Contact behavior was classed into socially exploring, aggressive, and defensive behavior. We analyzed the firing rates during the 3 different types of contact behavior. In the 15 rats in which multiunit activity was recorded in the PL, all rats showed socially exploring behavior. However, only 2 rats showed both aggressive and defensive behavior, 5 rats showed only aggressive behavior, and 3 rats showed only defensive behavior. No aggressive or defensive behavior was observed in 5 rats during the social interaction test. Multiple comparison procedures (Bonferroni test) revealed that the firing rate during aggressive behavior (8.9 ± 0.7 Hz, n = 7) was significantly higher than during socially exploring behavior (6.7 ± 0.4 Hz, n = 15) (*P* = 0.035) and defensive behavior (4.6 ± 1.0 Hz, n = 5) (*P* = 0.001) (Fig 7E). There was no significant difference between the firing rates during socially exploring behavior and defensive behavior (*P* = 0.082). In the 12 rats in which multiunit activity was recorded in the IL, all rats showed socially exploring behavior. However, only 1 rat showed both aggressive and defensive behavior, 3 rats showed only aggressive behavior, and 4 rats showed only defensive behavior. No aggressive or defensive behavior was observed in 4 rats during the social interaction test. The firing rates during socially exploring behavior, aggressive behavior, and defensive behavior were 5.1 ± 0.4 Hz (n = 12), 4.6 ± 0.6 Hz (n = 4), and 4.6 ± 0.4 Hz (n = 5), respectively. Multiple comparison procedures (Bonferroni test) showed that there were no significant differences among these firing rates (*P* = 1.000 each) (Fig 7F).

In the 15 rats in which multiunit activity was recorded in the PL, 11 showed both types of leaving behavior, in which the rat leaves the partner and the partner leaves the rat. Three rats showed only the leaving behavior in which the rat leaves the partner, and 1 rat showed only the leaving behavior in which the partner leaves the rat. Statistical analysis was performed in the 11 rats that showed both types of leaving behavior. There was no significant difference between the firing rates of PL neurons when the rat left the partner (5.9 ± 0.7 Hz) and when the partner left the rat (5.5 ± 0.6 Hz) (*P* = 0.582, paired *t* test, n = 11) (Fig 7G). In the 12 rats in which multiunit activity was recorded in the IL, 11 showed both types of leaving behavior. One rat showed only the leaving behavior in which the partner leaves the rat. In order to verify whether the factor of which rat leaves was significant in the increase in firing rate during leaving behavior, statistical analysis was performed in the 11 rats that showed both types of leaving behavior. The firing rate of IL neurons when the partner left the rat (7.6 ± 0.5 Hz) was significantly higher than when the rat left the partner (6.2 ± 0.3 Hz) (*P* = 0.009, paired *t* test, n = 11) (Fig 7H).

### Isolation-reared rats

#### Behaviors

After placement at opposite corners of the test box, the rats explored their environment and walked along the wall. Then, they showed approaching behavior. The mean latency until first approaching behavior was 24.0 ± 4.2 s, which was slightly longer than the group-reared rats, but not significantly different (*P* = 0.336; Student’s *t* test). The mean frequency of approaching behavior was 21.2 ± 0.9 s, which was significantly increased compared with the group-reared rats (*P* < 0.001). The mean total duration of approaching behavior was 38.2 ± 2.0 s, which was significantly increased compared with the group-reared rats (*P* < 0.001) (Table 2). The mean duration per one approaching behavior was 1.8 ± 0.1 s, which was not significantly different from the group-reared rats (*P* = 0.455). The mean frequency of contact behavior was 21.2 ± 0.9, which was significantly increased compared with the group-reared rats (*P* < 0.001). The mean total duration of contact behavior was 369.4 ± 20.8 s, which was significantly decreased compared with the group-reared rats (*P* = 0.006). The mean duration per one contact behavior was 19.0 ± 1.9 s, which was significantly shorter than the group-reared rats (*P* < 0.001). During contact behavior, aggressive and defensive behavior was observed rarely in isolation-reared rats. The mean frequency of leaving behavior was 20.8 ± 0.9, which was significantly increased compared with the group-reared rats (*P* < 0.001). The mean total duration of leaving behavior was 50.9 ± 2.6 s, which was significantly increased compared with the group-reared rats (*P* < 0.001). The mean duration per one leaving behavior was 2.4 ± 0.1 s, which was not significantly different from the group-reared rats (*P* = 0.296). The mean frequency of sole behavior was 21.8 ± 0.9, which was significantly increased compared with the group-reared rats (*P* < 0.001). The mean total duration of sole behavior was 441.5 ± 20.2 s, which was significantly increased compared with the group-reared rats (*P* = 0.045). The mean duration per one sole behavior was 21.2 ± 1.3 s, which was significantly shorter than that of the group-reared rats (*P* = 0.019).

**Table 2:** Duration and frequency of the behaviors of isolation-reared rats (n = 32) during the 15-min social interaction test.

	Total duration (s)	Frequency of episodes	Duration per episode (s)
Sole behavior	441.5 ± 20.2*	21.8 ± 0.9**	21.2 ± 1.3*
Approaching behavior	38.2 ± 2.0**	21.2 ± 0.9**	1.8 ± 0.1
Contact behavior	369.4 ± 20.8**	21.2 ± 0.9**	19.0 ± 1.9**
Leaving behavior	50.9 ± 2.6**	20.8 ± 0.9**	2.4 ± 0.1

Results are means ± SEM.

*P < 0.05

**P < 0.01 compared with the respective values of the group-reared rats (Table 1); Student’s *t* test.

These results indicated that the isolation-reared rats increased significantly the frequency of contact behavior and spent significantly less time in contact behavior compared with the group-reared rats, resulting in a significant decrease in the duration of contact behavior per contact.

#### Multiunit activity

Multiunit activity of the PL was recorded in 14 of 16 isolation-reared rats during the social interaction test; all recording sites were located in the PL, but clear multiunit activity was not recorded from 2 rats. Multiunit activity of the IL was recorded in 12 of 16 isolation-reared rats during the social interaction test; recording sites were located in the IL in 14 isolation-reared rats, and clear multiunit activity was not recorded from 2 of the 14 rats.

**Fig 8:**
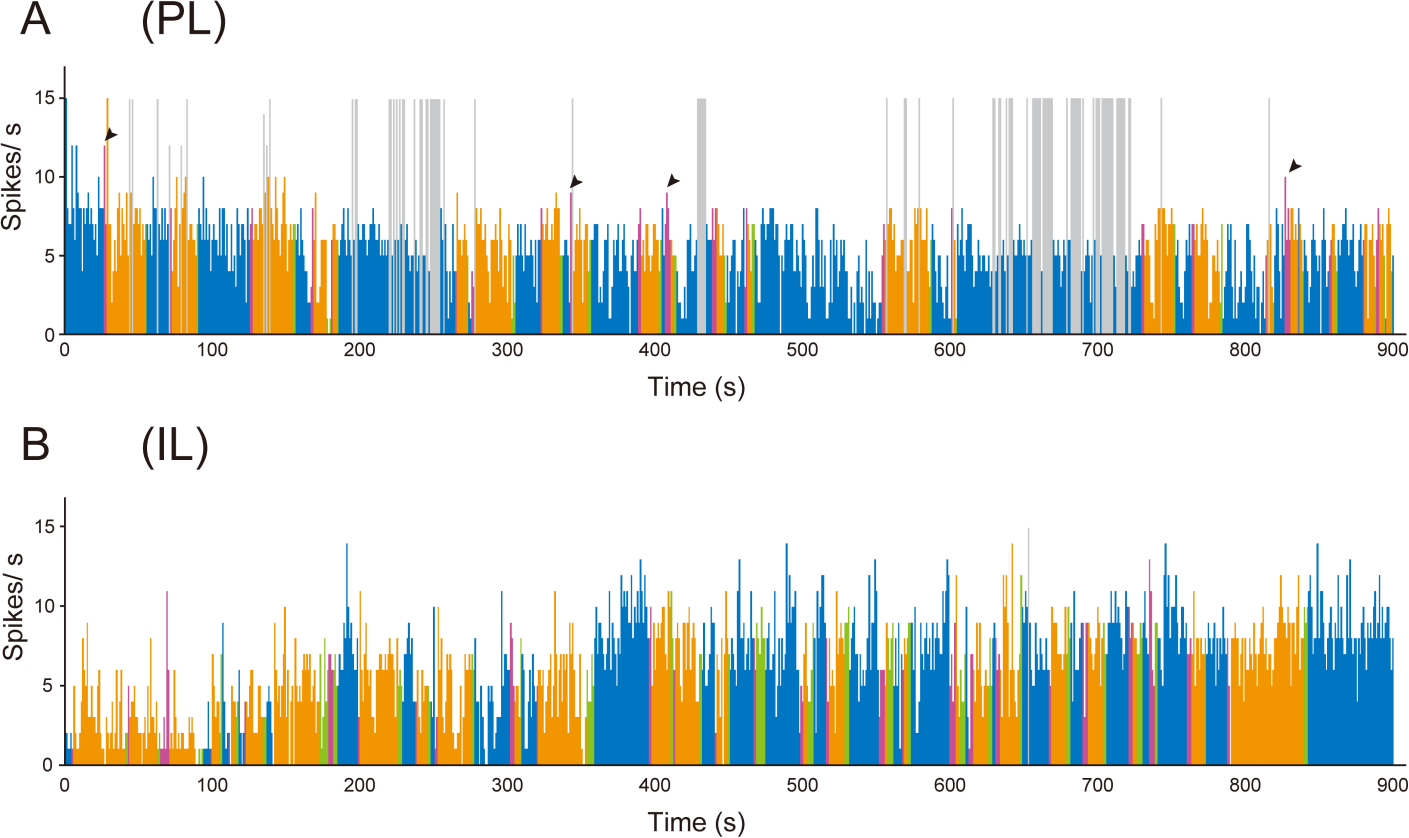
Histograms illustrating multiunit activity of PL and IL neurons of isolation-reared rats during the social interaction test. A: Multiunit activity recorded in the PL. The blue, red, orange, and green columns represent firing rate during sole, approaching, contact, and leaving behavior, respectively. The firing rate increases during approaching (red columns) behavior. The arrowheads indicate some of the instances when increased firing rates were observed during approaching behavior. The firing rate appears to increase during contact (orange columns) behavior. Mean firing rates during sole, approaching, contact, and leaving behavior in this rat were 5.1 Hz, 6.9 Hz, 6.3 Hz, and 4.4 Hz, respectively. B: Multiunit activity recorded in the IL. The blue, red, orange, and green columns represent firing rate during sole, approaching, contact, and leaving behavior, respectively. Mean firing rates during sole, approaching, contact, and leaving behavior in this rat were 7.5 Hz, 5.8 Hz, 5.6 Hz, and 6.6 Hz, respectively. The histogram bin size is 1 s. The gray columns indicate that multiunit activity was not discriminated from the artificial noise generated by sharp head shaking and self-grooming. When more than 2 behaviors were observed in a period of 1 column, the color of the behavior observed for the longest time is applied to the column.

Fairly stable firing was observed during sole behavior in PL and IL neurons (blue columns in Fig 8). In the PL neurons, firing was observed to increase during approaching and contact behavior (red and orange columns in Fig 8A); however, the increases seemed to be smaller than those in group-reared rats. Increases in firing during approaching and contact behavior were not observed in IL neurons (red and orange columns in Fig 8B). During leaving behavior, an increase in firing was not observed in PL neurons (green columns in Fig 8A) or IL neurons (green columns in Fig 8B).

**Fig 9:**
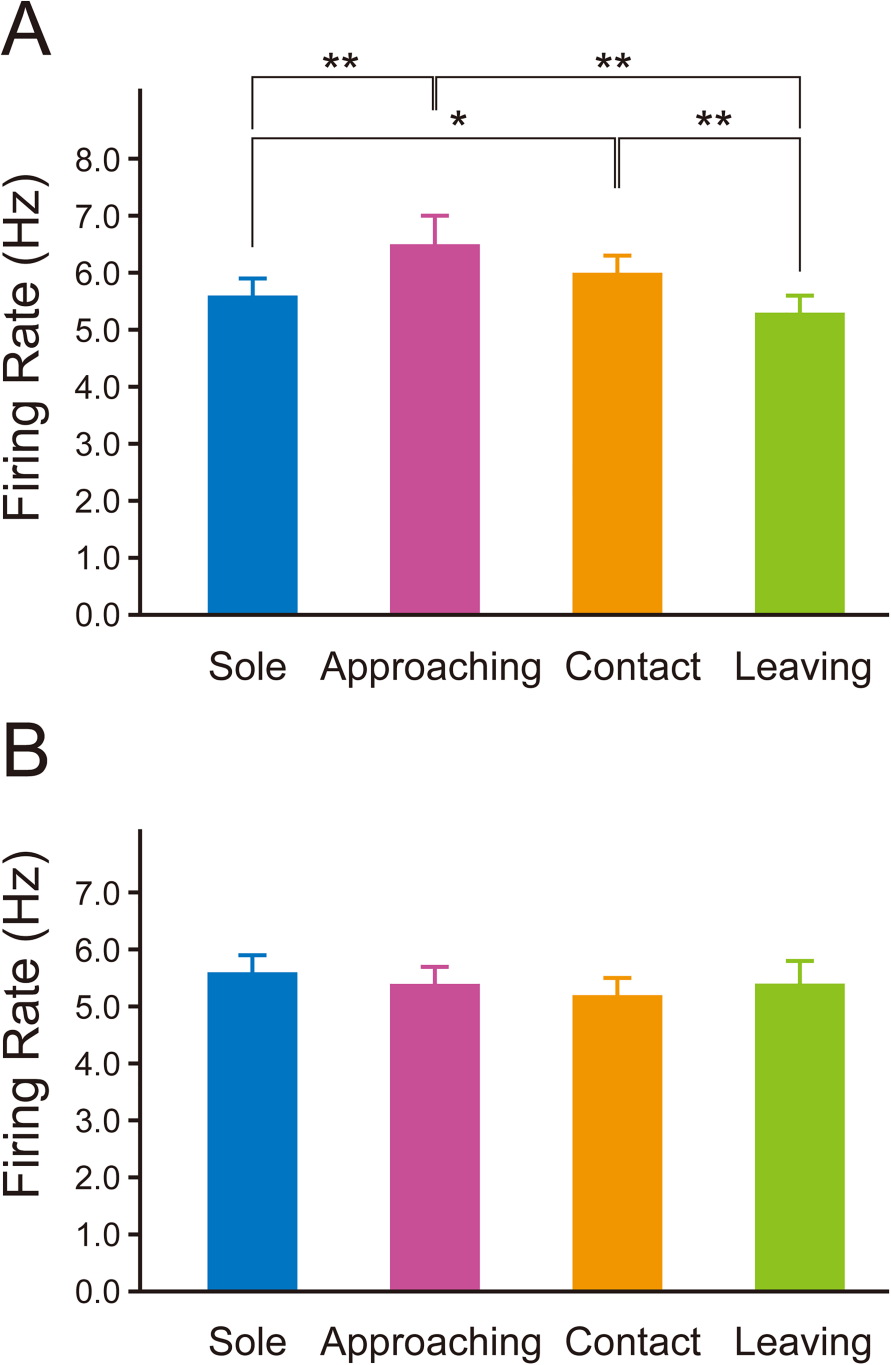
Firing rate of PL and IL neurons of isolation-reared rats in the social interaction test. A, B: Mean firing rates of PL neurons (n = 14) (A) and IL neurons (n = 12) (B) during sole (blue columns), approaching (red columns), contact (orange columns), and leaving (green columns) behavior. The firing rates of PL neurons during approaching and contact behavior were significantly higher than during sole and leaving behavior (A). No significant changes in the firing rate of IL neurons were observed during the 4 behaviors (B). Data represent means ± SEM. *P < 0.05, **P < 0.01; one-way repeated measures ANOVA with Bonferroni *post hoc* test.

In the 14 isolation-reared rats in which multiunit activity was recorded in the PL, the mean firing rate during sole behavior was 5.6 ± 0.3 Hz (range: 3.8–8.9 Hz). In the 12 isolation-reared rats in which multiunit activity was recorded in the IL, the mean firing rate during sole behavior was 5.6 ± 0.3 Hz (3.4–7.4 Hz). The mean firing rates of PL and IL neurons during approaching behavior were 6.5 ± 0.5 Hz (3.9–11.3 Hz) and 5.4 ± 0.3 Hz (3.2–6.9 Hz), respectively. The mean firing rates of PL and IL neurons during contact behavior were 6.0 ± 0.3 Hz (3.6–9.0 Hz) and 5.2 ± 0.3 Hz (3.7–6.7 Hz), respectively. The mean firing rates of PL and IL neurons during leaving behavior were 5.3 ± 0.3 Hz (3.4–7.6 Hz) and 5.4 ± 0.4 Hz (3.6–7.3 Hz), respectively. One-way repeated measures ANOVA revealed a significant difference among the firing rates of PL neurons during the 4 types of behavior (F_(3,39)_ = 8.049; *P* < 0.001). *Post hoc* comparisons (Bonferroni test) showed that the firing rate during approaching behavior was significantly higher than during sole behavior (*P* = 0.007) and leaving behavior (*P* = 0.003) and also that the firing rate during contact behavior was significantly higher than during sole behavior (*P* = 0.039) and leaving behavior (*P* = 0.005) (Fig 9A). There was no significant difference between the firing rates during approaching behavior and contact behavior (*P* = 0.162) and also between the firing rates during sole behavior and leaving behavior (*P* = 0.126). One-way repeated measures ANOVA revealed no significant difference among the firing rates of IL neurons during the 4 types of behavior (F_(3,33)_ = 0.525; *P* = 0.668) (Fig 9B).

**Fig 10:**
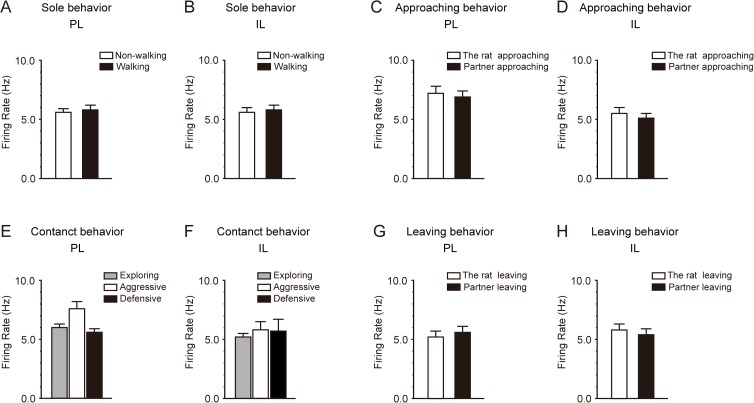
Firing rate of PL and IL neurons of isolation-reared rats during sole, approaching, contact, and leaving behavior. A, B: The firing rate of PL (A) and IL (B) neurons during sole behavior. Sole behavior was classed into non-walking (white columns) and walking (black columns) behavior. There were no significant differences between the firing rates during non-walking and walking behavior. C, D: The firing rate of PL (C) and IL (D) neurons during approaching behavior. Approaching behavior was classed into “the rat approaching the partner” (white columns) and “the partner approaching the rat” (black columns). There were no significant differences between the firing rates when the rat approached the partner and when the partner approached the rat. E, F: The firing rate of PL (E) and IL (F) neurons during contact behavior. Contact behavior was classified into 3 groups: socially exploring (gray columns), aggressive (white columns), and defensive (black columns). There were no statistical significant differences among these firing rates. G, H: The firing rate of PL (G) and IL (H) neurons during leaving behavior. Leaving behavior was classed into “the rat leaving the partner” (white columns) and “the partner leaving the rat” (black columns). There were no significant differences between the firing rates when the rat left the partner and when the partner left the rat. Data represent means ± SEM.

Sole behavior was classed into non-walking and walking behavior. In the PL, the firing rates during non-walking behavior and walking behavior were 5.6 ± 0.3 Hz and 5.8 ± 0.4 Hz, respectively, which were not significantly different (*P* = 0.363, paired *t* test, n = 14) (Fig 10A). In the IL, the firing rates during non-walking behavior and walking behavior were 5.6 ± 0.4 Hz and 5.8 ± 0.4 Hz, respectively, which were not significantly different (*P* = 0.417, n = 12) (Fig 10B). These results indicate that the neural activity of PL and IL neurons was not directly related to locomotion.

In the 14 isolation-reared rats in which multiunit activity was recorded in the PL, 11 showed both types of approaching behavior, in which the rat approaches the partner and the partner approaches the rat. One rat showed only the approaching behavior in which the rat approaches the partner, and the other 2 rats showed only the approaching behavior in which the partner approaches the rat. In order to verify whether the factor of which rat approaches was significant in the increase in firing rate during approaching behavior, statistical analysis was performed in the 11 rats that showed both types of approaching behavior. There was no significant difference between the firing rates of PL neurons when the rat approached the partner (7.2 ± 0.6 Hz) and when the partner approached the rat (6.9 ± 0.5 Hz) (*P* = 0.461, paired *t* test, n = 11) (Fig 10C). This result indicates that the factor of which rat approaches was not significant in the increase in the firing rate of PL neurons during approaching behavior. In the 12 rats in which multiunit activity was recorded in the IL, 9 showed both types of approaching behavior. Two rats showed only the approaching behavior in which the rat approached the partner, and another rat showed only the approaching behavior in which the partner approached the rat. Statistical analysis was performed in the 9 rats that showed both types of approaching behavior. There was no significant difference between the firing rates of IL neurons when the rat approached the partner (5.6 ± 0.4 Hz) and when the partner approached the rat (5.1 ± 0.4 Hz) (*P* = 0.400, paired *t* test, n = 9) (Fig 10D).

Contact behavior was classed into socially exploring, aggressive, and defensive behavior. Although aggressive and defensive behavior was observed rarely in isolation-reared rats, we analyzed the firing rates during the 3 different types of contact behavior. In the 14 isolation-reared rats in which multiunit activity was recorded in the PL, all rats showed socially exploring behavior. One rat showed both aggressive and defensive behavior, 2 rats showed only aggressive behavior, and 3 rats showed only defensive behavior. Eight rats did not show any aggressive or defensive behavior during the social interaction test. The firing rates during exploring, aggressive, and defensive behavior were 6.0 ± 0.3 Hz (n = 14), 7.6 ± 0.6 Hz (n = 3), and 5.6 ± 0.3 Hz (n = 4), respectively. Multiple comparison procedures showed that there were no significant differences among these firing rates (“exploring” vs. “aggressive,” *P* = 0.169; “aggressive” vs. “defensive,” *P* = 0.140; “exploring” vs. “defensive,” *P* = 1.000) (Fig 10E). In the 12 isolation-reared rats in which multiunit activity was recorded in the IL, all rats showed socially exploring behavior. However, no rat showed both aggressive and defensive behavior, 2 rats showed only aggressive behavior, and 3 rats showed only defensive behavior. Seven rats did not show any aggressive or defensive behavior during the social interaction test. The firing rates during exploring, aggressive, and defensive behavior were 5.2 ± 0.3 Hz (n = 12), 5.8 ± 0.7 Hz (n = 2), and 5.7 ± 1.0 Hz (n = 3), respectively. Multiple comparison procedures showed that there were no significant differences among these firing rates (*P* = 1.000 each) (Fig 10F).

In the 14 isolation-reared rats in which multiunit activity was recorded in the PL, 12 showed both types of leaving behavior, in which the rat leaves the partner and the partner leaves the rat. One rat showed only the leaving behavior in which the rat leaves the partner, and another rat showed only the leaving behavior in which the partner leaves the rat. Statistical analysis was performed in the 12 rats that showed both types of leaving behavior. There was no significant difference between the firing rates of PL neurons when the rat left the partner (5.2 ± 0.5 Hz) and when the partner left the rat (5.6 ± 0.5 Hz) (*P* = 0.381, paired *t* test, n = 12) (Fig 10G). In the 12 rats in which multiunit activity was recorded in the IL, 10 showed both types of leaving behavior. Two rats showed only the leaving behavior in which the rat leaves the partner. Statistical analysis was performed in the 10 rats that showed both types of leaving behavior. There was no significant difference between the firing rates of IL neurons when the rat left the partner (5.8 ± 0.5 Hz) and when the partner left the rat (5.4 ± 0.5 Hz) (*P* = 0.497, paired *t* test, n = 10) (Fig 10H).

## Discussion

This is the first study to demonstrate neural activity during social interaction. PL neurons showed increased firing during approaching behavior and also contact behavior, especially when the rat attacked the partner. It has been reported that a subset of mouse mPFC neurons elevate their firing rates when approaching a strange mouse [32] and that changes in the neurochemical conditions in the PL alter aggression in mice [33]. The present results support these reports. In contrast to PL neurons, IL neurons showed increased firing during leaving behavior, in which the firing rate when the partner left the rat was significantly higher than when the rat left the partner. Stimulation of the IL reportedly suppresses conditioned fear responses [34], inhibits aggressive behavior [35], and induces an antidepressant-like response [36]. In addition, the IL has been suggested to be responsible for the acquisition of stress resiliency [37]. The present result that increased firing of IL neurons was observed during leaving behavior may indicate that some relief from stress was induced during leaving behavior.

Recent studies on fear conditioning and extinction have proposed that the PL and IL have opposite effects on fear expression. Several studies using electrophysiological recordings have shown that the increased firing of PL neurons is correlated with sustained fear, whereas the increased firing of IL neurons is correlated with the recall of extinction [38,39]. A study using intracortical microstimulation of the PL and IL has shown that PL stimulation increases freezing behavior to a conditioned tone, whereas IL stimulation decreases freezing [16]. A study using inactivation of the PL and IL with a GABA_A_ receptor agonist has shown that the PL is involved in the expression of fear responses, whereas the IL is involved in their suppression [17]. Apart from fear expression, it has been proposed that the PL is essential for developing goal-directed response strategies, whereas the IL supports habit behavior in reward- and fear-related behavior (the “PL-go/IL-stop” model) [18], and that PL neural activity anticipates learning performance, whereas the IL lags in a switching memory strategy [40]. The opposite effects of the PL and IL could be applicable to the present forms of social interaction. PL neurons increased firing when a rat showed approaching behavior and also contact behavior, especially when a rat exhibited aggressive behavior to its partner, that is, when it acted actively toward the partner. Conversely, IL neurons increased firing when a rat exhibited leaving behavior, especially when its partner left the rat, that is, when the partner left on its own accord. The present findings in social interaction lead us to the following hypothesis that the PL is involved in active actions toward others, whereas the IL is involved in passive relief from cautionary subjects. Balanced activity between the PL and the IL may be important for the performance of suitable social interaction.

Isolation-reared rats showed an increased frequency and decreased duration of contact behavior in the present study. These results may be interpreted as that isolation-reared rats are interested in a novel partner but cannot continue contact behavior with the partner for a long period. Several studies have reported that isolation-reared rats show a decreased duration of social contact [26,30] and exhibit hyperactivity to a novel stimulus [23,24,41]. The present observations are in agreement with these reports. Aggressive behavior was induced during contact behavior in both group-reared and isolation-reared rats. It has been reported that play fighting is most frequent in young rats between P30 and P40 and then declines following puberty at approximately P60 [42,43], and is characterized by many role reversals in which rat is the attacker and which is the defender [44]. In the present study, the rats used for the social interaction test were at P65–P69 and did not show reversals in attacker and defender during an episode of aggressive and defensive behavior. Therefore, we consider the aggressive and defensive behavior observed in the present study as adult forms of these types of behavior. Statistical significant differences were not observed among the firing rates of exploring, aggressive, and defensive behavior in isolation-reared rats. This might be because not enough instances of aggressive and defensive behavior were observed for analysis in isolation-reared rats. It has been reported that social isolation increases aggressive behavior, in particular biting and boxing [45]. Such aggressive behavior was observed rarely in the isolation-reared rats of the present study. This differing finding in relation to the expression of contact behavior might be a result of strain differences between their Lister Hooded rats and our Sprague-Dawley rats.

PL neurons showed increased firing during approaching behavior and contact behavior in isolation-reared rats. These increases seemed to be smaller than those in group-reared rats, but they were still statistically significant. These results indicate that the increased firing of PL neurons, observed during approaching and contact behavior in group-reared rats, is preserved in isolation-reared rats. Conversely, IL neurons in isolation-reared rats did not show increased firing during leaving behavior, indicating that the increased firing of IL neurons observed during leaving behavior in group-reared rats is suppressed by isolation rearing. It is well known that hypo-activation of the IL impairs recall of extinction learning of conditioned fear [17,34,46] and is induced in patients with several neuropsychiatric disorders [8,9,47]. As hypothesized above, it may be important that the opposite effects of the PL and IL are balanced for the performance of suitable social interaction. The imbalance of neural activity between the PL and IL, in which the increased neural activity in the PL is preserved, whereas the increased neural activity in the IL is suppressed, may be one of the neural bases of isolation-induced behavior.

It has been reported that lesions of the orbitofrontal cortex produce significant deficits in social behavior [48–50]. Further studies are needed to clarify the neural activity of the orbitofrontal cortex in social interaction.

In conclusion, the present data demonstrate the neural activity of the PL and IL during social interaction and the influence of isolation rearing on this activity. PL neurons showed increased firing during approaching and contact behavior in group-reared rats, suggesting that they are involved in active actions toward others. In contrast, IL neurons showed increased firing during leaving behavior, especially when the partner leaves, suggesting that they are involved in passive relief from cautionary subjects. Isolation rearing induced an increased frequency and decreased duration of contact behavior, indicating that isolation-reared rats do not continue contact behavior for a long period. Isolation rearing showed differential effects on neural activity in the PL and IL. The increased neural activity in the PL, observed during approaching and contact behavior in group-reared rats, was preserved, whereas the increased neural activity in the IL, observed during leaving behavior in group-reared rats, was suppressed. The differential effects of isolation rearing on the neural activity of the PL and IL may be one of the neural bases of isolation-induced behavior.
